# Research on a CMOS-MEMS Infrared Sensor with Reduced Graphene Oxide

**DOI:** 10.3390/s20144007

**Published:** 2020-07-18

**Authors:** Shu-Jung Chen, Bin Chen

**Affiliations:** Department of Mechatronics Engineering, National Changhua University of Education, Changhua City 50074, Taiwan; d0751001@gm.ncue.edu.tw

**Keywords:** thermopile, reduced graphene oxide, characteristics measuring, CMOS-MEMS, infrared sensor

## Abstract

In this research, a new application of reduced graphene oxide (rGO) for a complementary metal-oxide-semiconductor (CMOS)-MEMS infrared (IR) sensor and emitter is proposed. Thorough investigations of IR properties including absorption and emission were proceeded with careful calibration and measurement with a CMOS thermoelectric sensor. The thermocouples of the sensor consist of aluminum and n-polysilicon layers which are fabricated with the TSMC 0.35 μm CMOS process and MEMS post-process. In order to improve the adhesion of rGO, a sensing area at the center of the membrane is formed with an array of holes, which is easy for the drop-coating of rGO material upon the sensing region. To evaluate the performance of the IR sensor with rGO, different conditions of the IR thermal radiation experiments were arranged. The results show that the responsivity of our proposed CMOS-MEMS IR sensor with rGO increases by about 77% compared with the sensor without rGO. For different IR absorption incident angles, the measurement of field of view shows that the CMOS-MEMS IR sensor with rGO has a smaller view angle, which can be applied for the application of long-distance measuring. In addition, characteristics of the proposed thermopile are estimated and analyzed with comparisons to the available commercial sensors by the experiments.

## 1. Introduction

Infrared (IR) elements with high thermal radiation and absorption capabilities can be used in many applications, such as temperature sensing and gas detection, among others. In addition to IR radiation sensing, they are also used as sources of IR radiation to provide active detection of signal sources in system applications. Generally, better radiation absorption efficiency will also have a better radiation intensity. In particular, in accordance with the current boom of miniaturized electronic devices and state-of-the-art MEMS (micro-electro-mechanical systems) technology using IR elements have also been increasingly reported [1,2,3,4,5,6,7,8].

In the past, one of the major issues of IR absorption technology had focused on the improvement of sensitivity for sensing thin films. There are some techniques that can enhance absorption for a specific range of IR thermal radiation by the interference type of ultrathin metal films or quarter-wavelength structures in [9,10] and a broadband of IR absorption by the structure of high-porosity or metal-black coatings [11,12,13,14,15]. These are reported as using polymer coatings filled with grains [16]. In addition, the exploration and research of new materials with high spectral absorption are constantly carried out.

Through design and improvement, absorption layers enhanced the sensing efficiency of thermal radiation in the above researches with the features as follows: (i) high absorption efficiency and uniform absorption spectrum, (ii) low heat capacity, (iii) high thermal conductivity, (iv) long-term stability and reproducibility and (v) deposition process of material compatible with the processes of fabrication of the element [17,18,19,20,21,22]. It highlights the main characteristics to be considered for the development of IR thermal radiation-sensing thin films.

Beyond these technologies, in recent years, new opportunities for sensing materials are offered due to recent advances in research and the invention of novel materials [23,24]. In 2013, Mikyung Lim et al., coated a monatomic layer of graphene on doped silicon substrates and found that it could increase the radiant heat flux between the two plates [2]. Here, graphene, with its sp^2^-hybridized, honeycomb, two-dimensional carbon lattice consisting of conjugated hexagonal cells, shows extraordinary properties.

In comparison with graphene, reduced graphene oxide (rGO) has an incomplete structure, involving abundant oxygen functional groups located in the basal plane (forming C–O–C bonds) at the edges of defective areas. At the time when rGO is receiving IR radiation, the free electrons localized near the oxygen are susceptible to radiation disturbances. This causes resonant induction which leads to the large enhancement in IR absorption [25].

In 2014, Haifeng Liang reported a mid-IR photoresponse of rGO with a responsivity of 1 A/W by a phototransistor based on a graphene double-layer heterostructure [26,27]. In 2016, the Parikshit Sahatiya team used graphene oxide and reduced graphene oxide (synthesized rGO) drop-coated by the Hummer method to make portable sensing films for temperature sensing [28]. In 2018, Abid et al., reported a facile and cost-effective approach to develop a self-standing rGO film-based optical sensor and its low-temperature performance analysis [29]. However, most of these studies are based on the sensing technology of graphene oxide in the quantum detector, which is devoted to the enhancement of the quantum efficiency and conductivity of quantum detectors.

In recent decades, with the development of complementary metal-oxide-semiconductor (CMOS)-MEMS technology, thermopile IR sensors can be very small in size, mass and volume, with low cost, very low power consumption and high sensitivity, and can be manufactured in batches [30,31]. Our research suggests that it will be more practical in thermal IR sensor applications and compatible with standard CMOS processes and more simplified signal processing. Due to the maturity of the processes and being compatible with standard CMOS technology, we devoted the promotion of IR elements to using CMOS-MEMS techniques which have proven particularly effective in the development of system on a chip (SOC) technology [32,33,34]. In summary, we give a new approach to improve the sensitivity of a CMOS-MEMS IR sensor with drop-coated rGO to achieve high thermal radiation absorption. The work focuses on the investigation of absorption characteristics and infrared emission of rGO, and a specific thermopile is adapted which plays a role as a test platform in the experiment. Then, through measurement and analysis, the relevant characteristics of the different sensors are compared to verify its excellent thermal radiation absorption and emission characteristics.

## 2. Design and Fabrication

**Figure 1:**
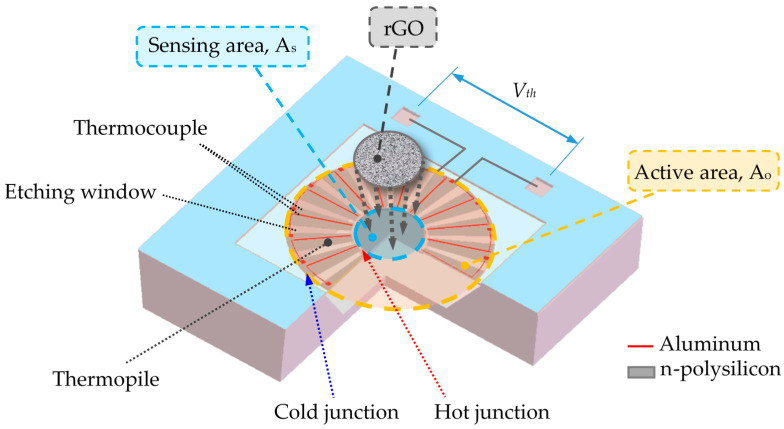
Schematic drawing of the complementary metal-oxide-semiconductor (CMOS)-MEMS thermopile.

The proposed CMOS-MEMS IR sensor includes a floating membrane with a sensing area where the sensing material was drop-coated. This CMOS-MEMS thermopile was designed using the materials from the CMOS layers of aluminum and n-polysilicon as the thermoelectric elements. The schematic drawing of the CMOS-MEMS thermopile is shown in Figure 1. Here, the thermopile, based on the Seebeck effect, was used for the monitoring of IR radiation sensing or emitting. rGO was drop-coated on the center of the thermopile to absorb the thermal radiation. In this manner, the IR radiation influences the difference in temperature between the hot and cold junctions in the thermocouples, which in turn leads to a small variation in voltage. The CMOS-MEMS thermopile comprised multiple thermocouples (in series), which generated an output signal by which to measure the absorption of IR radiation. The design and fabrication of the CMOS-MEMS thermopile are presented below. 

### 2.1. Design and Fabrication

The verification chip, CMOS-MEMS thermopile, required for our experiments was fabricated using a TSMC 0.35 μm CMOS standard process. There are 64 pairs of thermocouples around the center of the sensing membrane. The thermocouples were arranged in a circularly symmetrical array to obtain a large active area for IR radiation sensing. To maximize the signal-to-noise ratio (SNR), the patterning of the N-polysilicon and aluminum were designed carefully and evaluated to minimize resistance, Johnson’s noise and thermal conductance. The length and width of the thermocouple extending outward are approximately 300 and 25 μm, respectively. The width of the metal is 3 μm. The sensing area with an array of holes was placed in the center of a thermopile structure in which the radius of holes is 6 μm with a space distance of 2 μm.

**Figure 2:**
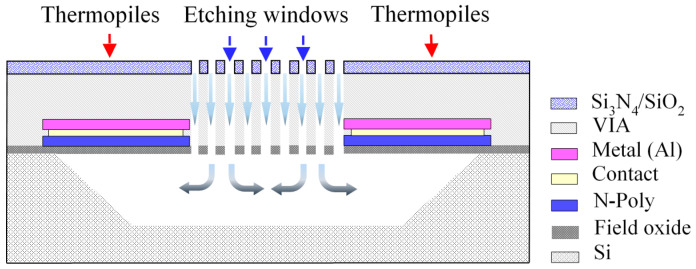
Schematic drawing of the cross-section of the thermopile with the MEMS post-process.

To remove the silicon substrate beneath the membrane efficiently, there are etching windows (length = 280 μm; average width = 5 μm) of a slim-long type situated between each two thermocouples which allow the etchant to flow through. After the CMOS standard process was completed, the MEMS fabrication process including an isotropic RIE (Reactive-ion etching) and wet etching processes was also used to perform the substrate etching. The cross-section of the thermopile is shown in Figure 2, which illustrates the steps of the MEMS fabrication process. Then, the rGO sensing material was used to drop on the sensing area.

**Figure 3:**
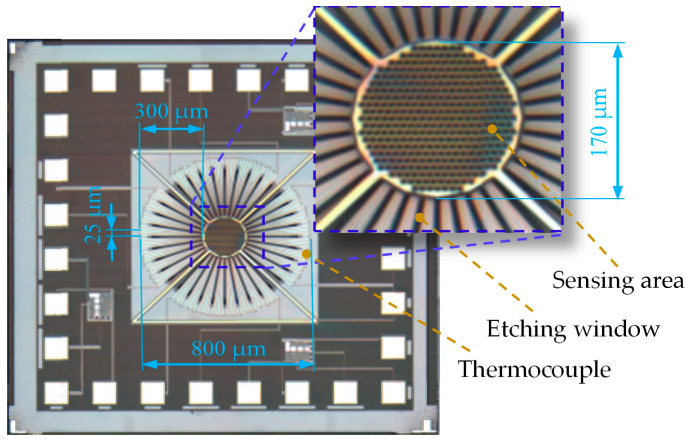
Microscope photograph of the proposed CMOS-MEMS thermopile.

The membrane on the sensor with a diameter of 800 μm was defined as the active area *A_o_* of the thermopile which includes the central sensing area *A_s_* with a diameter of 170 μm and the outer area distributed with thermocouples. The resistance of the thermopile is about 11.36 kΩ for all the thermoelectric elements connected in series. Figure 3 shows the microscope photograph of the proposed CMOS-MEMS thermopile with an array of holes.

### 2.2. Post-Processing

In preparation for the sensing material, rGO powder and NMP (*N*-Methyl-2-pyrrolidone, C**_5_**H**_9_**NO) were the primary materials. Firstly, 0.16 g of rGO powder was added to 12 g NMP, followed by ultrasound, oscillating for 15 min to averagely disperse the rGO molecule in NMP. Then, the solution was stirred with the magnet mixer for 300 rpm, 20 min, making the preparation of the sensing material complete.

**Figure 4:**
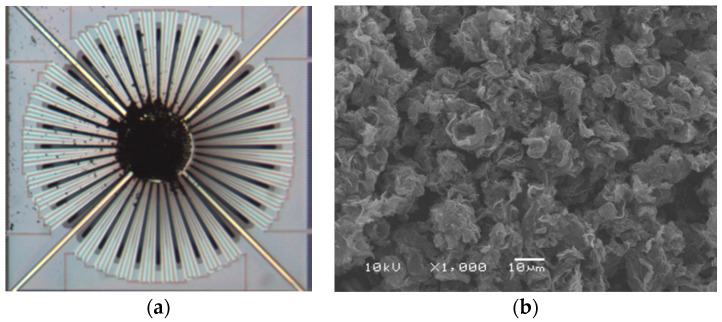
(**a**) The proposed CMOS-MEMS thermopile after drop-coating; (**b**) An SEM for the topology of rGO.

After the drop-coating of rGO, the thermopile was dried in a vacuum chamber for 20 min. The photograph of the final CMOS-MEMS thermopile with rGO and an SEM for the topology of rGO are shown in Figure 4. It is shown that the rGO reveals a rough and porous surface with the grain size of 10 μm to 20 μm in the SEM image. A rough surface is supposed to give a high emissivity which enhances the absorption property for IR elements.

## 3. Characteristics Measurement and Analysis

Thorough investigations include measurements of IR thermal radiation absorption and emission to explore the effect of adding rGO on the sensing thin film. Then, the experiments and analyses of frequency response and noise were arranged to realize the characteristics of the proposed CMOS-MEMS thermopile with rGO. Finally, the measurements of the incident angle for IR sensing were also carried out.

**Figure 5:**
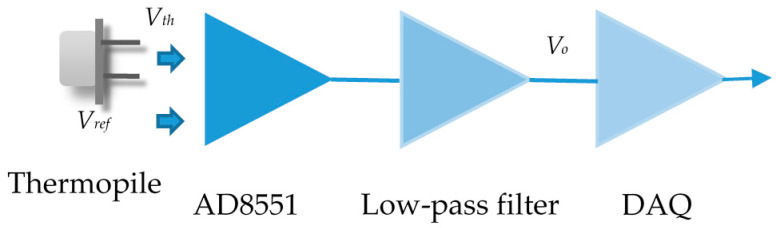
Amplification circuit for the thermopile.

**Figure 6:**
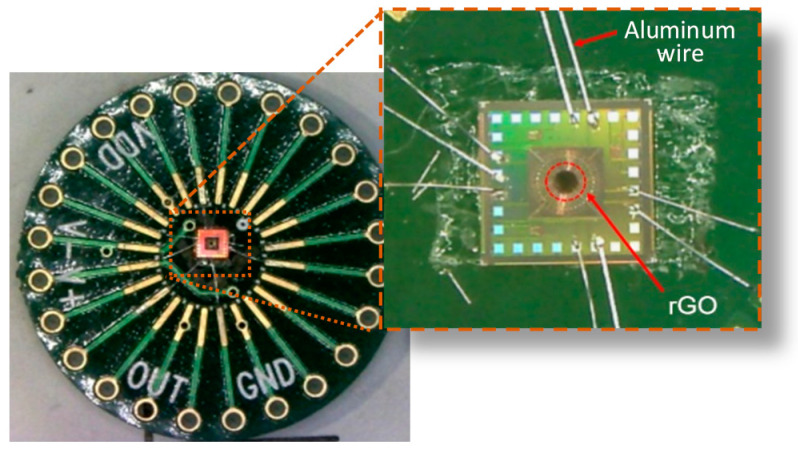
The measured circuit board with the CMOS-MEMS thermopile.

The function block of the measurement circuit includes a chopper amplifier (AD8551), low-pass filter and data acquisition circuit (DAQ), which is shown in Figure 5. The original output signal of the thermopile denoted as *V_th_* was amplified based on a reference voltage *V_ref_*, which was set by the resistor divider and the buffer. The non-inverting amplification was adopted with a gain of 620. The measured circuit board with the thermopile is shown in Figure 6.

The original output signal of the thermopile *V_th_* can be derived from the output voltage *V_o_* of the measurement circuit and the circuit parameters including the gain and the reference voltage of the amplification circuit.

Under the experimental arrangements of transient signal acquisition and frequency modulation, several characteristics of the thermopile will be further discussed. Then, the difference in characteristics between the thermopile with and without rGO can be compared in the following sections.

### 3.1. Thermal Radiation Absorption Characteristic

#### 3.1.1. Measurement of Thermal Radiation Absorption

**Figure 7:**
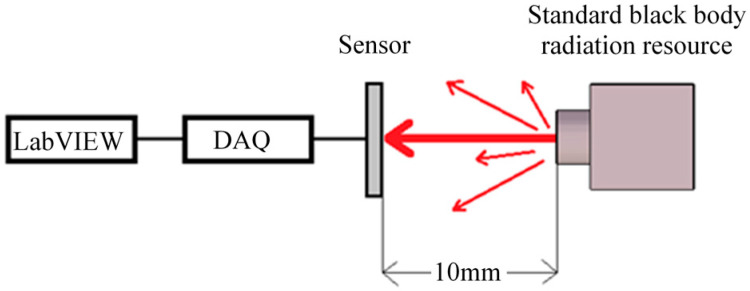
Measurement of radiation absorption for the sensors.

The measurement of thermal radiation absorption for the sensors was proceeded as the setup of the measurement shown in Figure 7. A temperature well-controlled standard black body (HOTECH Model 390) with an aperture diameter of 60 mm was used as the IR radiation source. The thermopile under test was placed at a 10 mm distance in the front of the black body.

**Figure 8:**
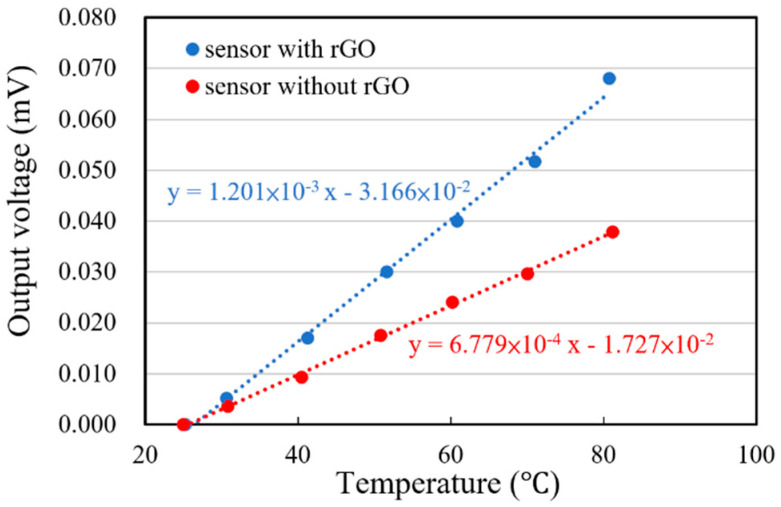
Output voltage of the sensors vs. target temperature from the black body.

To analyze and calibrate the responsivity of the thermopiles with and without rGO, the experiment was carried out at a room temperature of 25 °C and compared to a standard infrared sensor, OTP-N537F2. The target temperatures from the standard black body range from 25 to 80 °C for the measurement. The output voltages *V_o_* were acquired and converted to digital data by DAQ (data acquisition) and then recorded by the LabVIEW software. After data manipulations, the original output signals *V_th_* of the thermopiles were derived and recorded in Figure 8.

#### 3.1.2. Analysis of Thermal Radiation Absorption

The precise measurement of thermal radiation is based on that the exchanges of radiation between the black body, the sensor and the environment reach a thermal equilibrium. To understand the performance of using the thermopile with rGO to sense thermal radiation, the experimental results in Figure 8 were used for further analysis.

In addition to the thermal radiation emitted by the standard black body, the thermal radiation from the sensor plays the key issue for influencing the output signal of the thermopile in this study. Due to the stable thermal equilibrium, the mutual radiation of the sensor and the environment cancel each other, which is ignored here. Moreover, emissivity is also an important factor to consider on the surface for thermal radiation. Emissivity is the ratio of the thermal radiation from a surface of the material to the radiation from a perfect emitter, known as a black body, at the same temperature and wavelength and under the same viewing conditions.

Based on the Stefan–Boltzmann law, the net power ∆*Φ* of the thermopile includes absorption and emission between the black body and the sensor, as shown in Equation (1).

(1) $${\Delta \Phi =g}_{b}A_{b}A_{o}\varepsilon_{b}\varepsilon_{s}{\sigma T}_{b}^{4}-g_{s}A_{b}A_{o}\varepsilon_{b}\varepsilon_{s}{\sigma T}_{s}^{4}$$

where *g_b_* and *g_s_* refer to the geometrical parameters of the optical path from the black body to the sensor and from the sensor to the black body, respectively; *A_b_* is the radiation area of the black body; *A_o_* is the active area of the sensor; and *ε* and *T* indicate the emissivity and the absolute temperature. The subscript *b* and *s* are denoted as the corresponding parameters of the black body and the sensors. *σ* is the Stefan–Boltzmann constant.

For comparing the emissivity of the sensing area on the thermopile with rGO to the one without rGO, the thermal behavior of the sensor can be described as the following equation:

(2) $$H\frac{{dT}_{s}}{dt}+G\left(T_{s}-T_{a}\right)=\Delta \Phi$$

where *H* and *G* indicate the heat capacity and total thermal conductance of the sensor, respectively, whereas *T_a_* refers to the ambient temperature. Based on the Seebeck effect, the original output voltage *V_th_* of the thermoelectric sensor can be derived from the difference in temperature Δ*T* = *T_s_* − *T_a_*. In a steady-state condition, Equation (2) is reformulated accordingly as follows:

(3) $$V_{th}=N\cdot \Delta S\cdot \frac{\Delta \Phi }{G}$$

where *N* is the number of pairs of the thermocouples on the thermopile and Δ*S* is the difference between the Seebeck coefficients of the two types of material used in the thermocouple. The derivative of *V_th_* with respect to *T_b_* yields the following.

(4) $$\frac{{dV}_{th}}{{dT}_{b}}=N\cdot \Delta S\cdot \frac{d\Delta \Phi }{G\cdot {dT}_{b}}$$

The values of (*dV_th_*/*dT_b_*)*_g_* and (*dV_th_*/*dT_b_*)*_n_* from Figure 8 are used in Equation (5), where the ratio *r* is defined. Here, the subscripts *g* and *n* have been introduced to indicate the corresponding parameters of the thermopiles with and without rGO, respectively.

(5) $$r=\frac{{\left(\frac{{dV}_{th}}{{dT}_{b}}\right)}_{g}}{{\left(\frac{{dV}_{th}}{{dT}_{b}}\right)}_{n}}=\frac{{1.201\times 10}^{-3}}{{6.779\times 10}^{-4}}=1.77$$

It is necessary to consider the irradiation on the whole active area *A_o_*, including the areas with and without rGO, shown in Figure 1, for evaluating the difference of the emissivity between the thermopiles with and without rGO. Therefore, Equation (4) is rewritten by introducing the active area *A_o_* and the sensing area *A_s_* according to Equation (1) as follows.

(6) $$\frac{{\left(\frac{{dV}_{th}}{{dT}_{b}}\right)}_{g}}{{\left(\frac{{dV}_{th}}{{dT}_{b}}\right)}_{n}}=\frac{\varepsilon_{g}\cdot A_{s}+\varepsilon_{n}\cdot \left(A_{o}{-A}_{s}\right)}{\varepsilon_{n}\cdot A_{o}}$$

where *ε_g_* and *ε_n_* are the emissivity of the sensing area on the thermopiles with and without rGO, respectively. Hence the ratio of the emissivity of the sensing area on the thermopiles with and without rGO in the thermal radiation absorption experiment is derived by substituting *r* in Equation (5), which is governed in Equation (7).

(7) $$\frac{\varepsilon_{g}}{\varepsilon_{n}}=\frac{\left(r-1\right)\cdot A_{o}+A_{s}}{A_{s}}=\left(r-1\right)\cdot \frac{A_{o}}{A_{s}}+1$$

The active area *A_o_* and the sensing area with rGO *A_s_* were designed and patterned to have an *A_o_*/*A_s_* ratio of 6.25. In thermal radiation absorption, the ratio of emissivity of the sensing area on the thermopile with rGO to the one without rGO is 4.92 from Equation (7), consequently. The method of drop-coated rGO on the thermopile element we proposed in this study can significantly improve the output signal of the thermopile by increasing the emissivity.

### 3.2. Thermal Radiation Emission Characteristic

#### 3.2.1. Measurement of Thermal Radiation Emission

**Figure 9:**
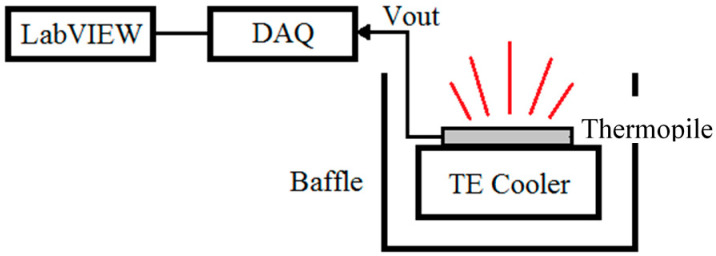
Measurement of radiation emission for the thermopiles.

**Figure 10:**
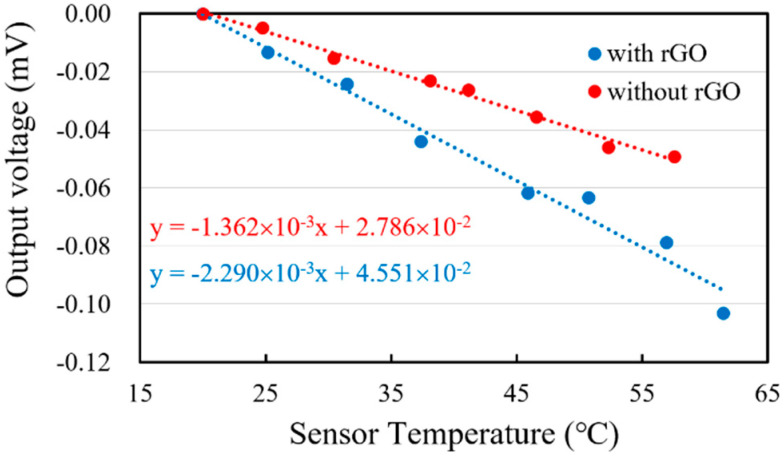
Output voltage vs. temperature of the sensors.

The investigation of the emissivity of a thermal emitter has attracted growing interest, with a view toward a new generation of thermal emission devices. Therefore, the experiment for thermal radiation emission characteristics of the thermopiles with and without rGO was designed at a fixed ambient temperature, 25 °C, to explore the radiation emission efficiency as an IR micro-emitter. The IR emission of rGO was proceeded based on the same structure of the thermopile, which plays the role of the test platform. With a well temperature-controlled TE cooler, the IR radiation is emitted from the sensing area of rGO to the environment for an initial exploration, as shown in Figure 9. When the TE cooler was driving, a calibrated thermistor was used to monitor the temperature for the heating sensor. The measurement results show that the original output signals from the thermopile with rGO are lower than the thermopile without rGO, which are shown as Figure 10. It means that the thermal radiation from the thermopile with rGO is scattered sooner than the thermopile without rGO.

#### 3.2.2. Analysis of the Thermal Radiation Emission

To investigate the performance of using the thermopile with rGO as a thermal radiation source, the experimental results in Figure 10 were used for further analysis. When measuring the radiation emission of the thermopile, the heated thermopile emits thermal radiation to the environment. However, the surrounding environment also emits thermal radiation to the thermopile. Since the temperature of the environment is lower than the heated thermopile, the output voltage of the thermopile is negative, as shown in Figure 10. The net power of the thermopile including emission and absorption between the sensor and environment is given by Equation (8).

(8) $${\Delta \Phi =g}_{a}A_{a}A_{o}\varepsilon_{a}\varepsilon_{s}{\sigma T}_{a}^{4}-g_{s}A_{a}A_{o}\varepsilon_{a}\varepsilon_{s}{\sigma T}_{s}^{4}$$

The subscript of *a* is used for the corresponding parameters of the surrounding environment. Using Equation (2) to Equation (7), we can also derive the ratio of the emissivity in the thermal radiation emission experiment. However, the derivative of *V_th_* was acquired with respect to *T_s_* in the thermal radiation emission experiment.

By the measured data in Figure 10, the values of (*dV_th_*/*dT_s_*)*_g_* and (*dV_th_*/*dT_s_*)*_n_* are also substituted into Equation (9) to derive the ratio *r* for the thermal radiation emission as follows.

(9) $$r=\frac{{\left(\frac{{dV}_{th}}{{dT}_{s}}\right)}_{g}}{{\left(\frac{{dV}_{th}}{{dT}_{s}}\right)}_{n}}=\frac{-{2.290\times 10}^{-3}}{-{1.362\times 10}^{-4}}=1.68$$

Therefore, the ratio of emissivity of the sensing area on the thermopiles with rGO to the thermopile without rGO is 4.41, from Equation (7), in the thermal radiation emission. This result is an approach to that in thermal radiation absorption. In general, the spectral absorption of SiO_2_ is reported at an average of 0.2 for the IR wavelength from 5 μm to 14 μm [35].

### 3.3. Measurement and Analysis of the Sensor

#### 3.3.1. Analysis of Responsivity

Responsivity is an important characteristic to realize the sensitivity of a thermoelectric sensor. In general, the responsivity of the IR sensor can be calculated by Equation (10).

(10) $$R_{s}=\frac{{dV}_{th}}{d\Phi }$$

where *Φ* is the radiated power that reaches the sensor. However, the responsivity of the thermoelectric sensor is not easy to obtain directly. To calibrate the responsivity of the proposed thermopile with rGO, according to Equation (10) and the Stefan–Boltzmann law, the relation between the responsivity of the standard thermopile OTP-N537F2 and of the proposed thermopile by Equation (11) is obtained as follows.

(11) $$R_{s}{=R}_{OTP}\times \frac{A_{OTP}}{A_{s}}\times \frac{{\left(\frac{{dV}_{th}}{{dT}_{b}}\right)}_{s}}{{\left(\frac{{dV}_{th}}{{dT}_{b}}\right)}_{OTP}}$$

where the subscript OTP is denoted as the corresponding parameters of the standard thermopile. The sensing areas of the proposed thermopile and the standard thermopile are with a radius of approximately 85 and 272.5 μm, respectively. The values of *dV_th_*/*dT_b_* from the different sensors are derived from Figure 8. (*dV_th_*/*dT_b_*)*_OTP_* from the standard thermopile is 8.5 × 10^−2^ mV/°C. (*dV_th_*/*dT_b_*)*_g_* and (*dV_th_*/*dT_b_*)*_n_* from the thermopiles with and without rGO are 1.201 × 10^−3^ mV/°C and 6.779 × 10^−4^ mV/°C, respectively. The responsivity of the standard thermopile is 87 V/W. After substituting the above values to Equation (11), the responsivity of the thermopiles with and without rGO are estimated, which are 14.522 and 8.197 V/W, respectively. The results show that the responsivity of the thermopile with rGO can be improved by about 77 % compared with the thermopile without rGO.

#### 3.3.2. Measurement and Analysis of Frequency Response and Thermal Time Constant

**Figure 11:**
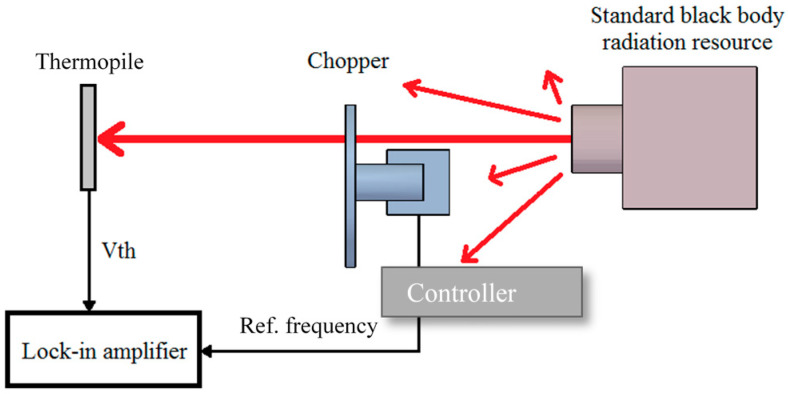
Measurement of the frequency response for the thermopiles.

The experiment was executed to get the frequency response of the thermopiles with and without rGO, in which the setup of the measurement is shown in Figure 11. The thermal radiation with a fixed temperature of 200 °C was created from the standard black body radiation source. The chopper was set up between the thermopile and the black body source. The frequency of the chopper was adjusted by the controller which also provided a reference frequency to the PLL amplifier so that the weak sensing signal from the thermopile was acquired.

**Figure 12:**
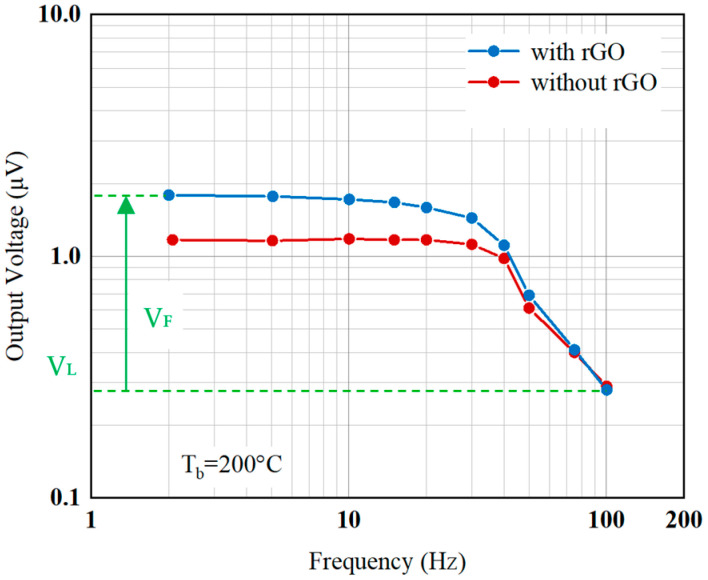
Frequency response of the thermopiles with and without rGO.

It is observed that the original output signals of the thermopile with rGO at different frequencies are also higher than that of the thermopile without rGO, as expected in Figure 12. Note that the thermopile with rGO shows a slower response in the frequency response. Since the thermal time constant is determined by the thermal capacity and thermal conductivity of the film on the thermopile. It may be caused by the drop-coating of rGO which increases the mass of the film on the thermopile, i.e., it has a larger heat capacity value. At this time, although rGO is a material with good thermal conductivity, the ratio of the increase in thermal conductivity is relatively smaller than that in heat capacity, so the thermopile with rGO has a larger time constant.

In this experiment, the frequency response of the thermopile output signals is derived as the increases in voltage from the lowest baseline voltage (*V_L_*) shown in Figure 12. Therefore, the original output signals of the thermopiles at the flat band (*V_F_*) with and without rGO are 1.51 and 0.88 μV, respectively. The output voltage of the thermopile with rGO is 71.6 % higher than that of the thermopile without rGO, which nearly coincides with the results of Equation (5) and Equation (9). At the same time, the cut-off frequencies of the thermopiles with and without rGO in the frequency response of its output signals are 48.0 and 68.5 Hz, respectively. The cut-off frequency of the thermopile without rGO is 29.9 % higher than that of the thermopile with rGO. The corresponding time constants of the thermopiles with and without rGO were calculated as 3.32 × 10^−3^ s and 2.32 × 10^−3^ s, respectively. The thermal time constant of the thermopile with rGO shows an increase of 43.1 % compared with the thermopile without rGO. Since the thermal time constant depends on the heat capacity, H, it will therefore be affected by the volume of rGO in the drop-coating.

#### 3.3.3. Measurement of Noise

Noise exists in electronic devices, which affects the accuracy of the thermopile during measurement. With the acquisition of noise, more characteristics of the thermopiles with and without rGO could be investigated and evaluated. In order to further analyze the signal and noise of the sensor, the noise spectrum was measured. Through a lock-in amplifier, the influence of noise on the thermopile at each frequency was known in the following experiment. In the noise measurement, the thermopile was sealed to isolate the interference of environmental factors. Before measuring, the linear filter needed to be set to filter 60 Hz noise. The alternating current as a tracking signal from a function generator (AFG 3101) was delivered to the PLL amplifier (7225 DSP Lock-in Amplifier). According to this reference frequency, the original output signals of the thermopiles were recorded by the PLL amplifier which are the real noises of the elements.

**Figure 13:**
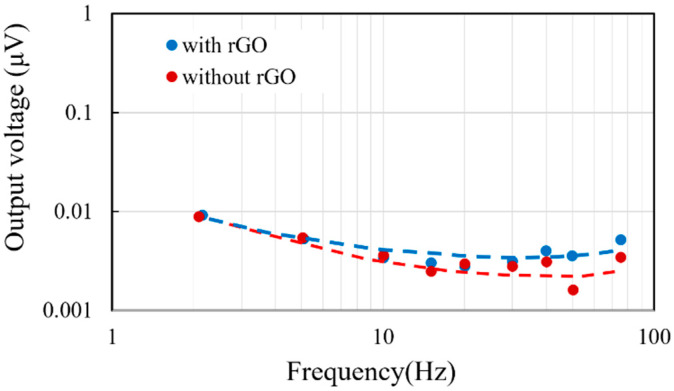
Frequency response of the average noise from the thermopiles with and without rGO.

The results of several measurements from the thermopile with and without rGO are shown in Figure 13. All the signals of the noises are below 0.1 μV and it shows a nearly flat level around 30 nV to 35 nV within 10 Hz to 100 Hz. At the same time, the difference in noise between the thermopiles with and without rGO is not too significant.

#### 3.3.4. Analysis of SNR

In addition to the responsivity, in IR sensors, we can also evaluate the characteristics of the device through some sensor performance parameters, such as noise, SNR, noise equivalent power (NEP) and normalized detectivity (D*). Here, the noises of the thermopile with and without rGO were estimated at room temperature firstly. Usually, to calculate performance parameters of sensors, Johnson noise will be adopted directly as the noise of the IR sensor. Johnson noise is the main source of the noise of the thermopile which can be obtained using Equation (12).

(12) $$V_{N}=\sqrt{4kTR\Delta f}$$

where *k* is the Boltzmann constant and ∆*f* is the unit frequency bandwidth of Johnson noise. A total resistance *R* of the thermopiles is 11.36 kΩ. Although rGO was drop-coated on the surface of the thermopile, the noise of the sensor is mainly from the contribution of the resistance of the thermopile with or without rGO, and its value is about 13.716 $\mathrm{nV}/\sqrt{H_{Z}}$ for both.

The original output signals measured at room temperature were divided by Johnson noise in rms with a frequency bandwidth of 1 Hz, and the SNRs of the thermopiles with and without rGO are 291.54 and 364.43, respectively. The SNR of the thermopile with rGO increases about 25 % relative to the thermopile without rGO.

#### 3.3.5. Figure of Merit of the Sensor

After the noise was acquired, more characteristics of the thermopiles with and without rGO were analyzed and compared. NEP can be calculated by dividing the responsivity results obtained in the previous analysis by Johnson noise. The detectivity of the thermopile is the reciprocal of the NEP. Normalized detectivity D* indicates detectivity regardless of the influences of the sensing area and bandwidth as the following equation:

(13) $$D^{*}=\frac{\sqrt{A_{s}\Delta f}}{NEP}$$

Among them, normalized detectivity D* is the most important performance index for evaluating IR sensor elements. The D* of the thermopile with rGO is 1.77 times that of the thermopile without rGO. Figure of merits of the thermopiles with and without rGO are summarized in Table 1.

#### 3.3.6. Measurement and Analysis of Incident Angle of IR Radiation

**Figure 14:**
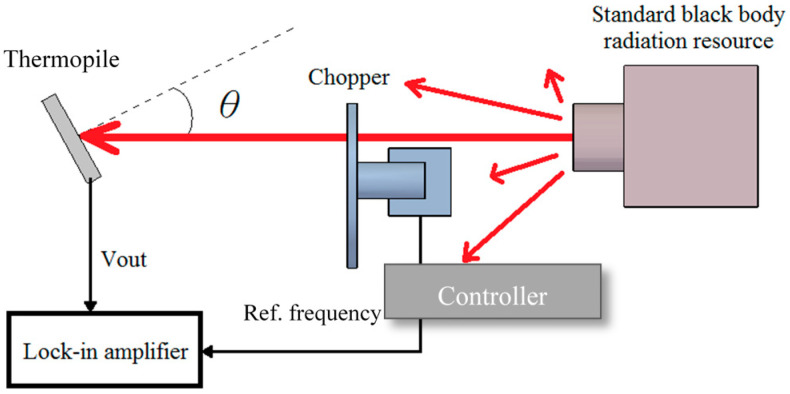
Measurement of IR incident angle for the thermopiles with and without rGO.

The influence of the different incident angle of IR radiation on the thermopiles was also investigated. The experiment setup is shown in Figure 14. Thermal radiation was generated by the standard black body which had a distance of 200 mm from the thermopile. The chopper was placed in the middle of them, and a reference frequency from the controller of the chopper was delivered to the PLL amplifier. The thermopile was fixed on the rotator, and through the rotating device, we can adjust the different deflection angles relative to the standard black body for measurement. The original output signals of the thermopiles with and without rGO were recorded from 0 to 45 incident angles, respectively.

**Figure 15:**
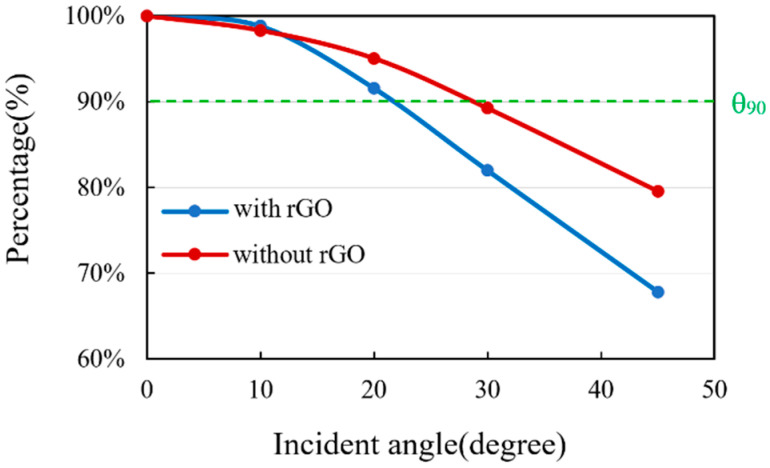
Output signal comparison vs. incident angle.

To compare the effect of the different incident angles of IR radiation to the output signals of the thermopiles with and without rGO, the measurement results are shown in Figure 15. 

A view angle *θ*_90_ is defined for the output signal *V_th_* decrease to the 90 % of the maximum of the output signal. The *θ*_90_ for the thermopiles with and without rGO is 22.5° and 30.5°, respectively.

The signal intensity of the thermopile with rGO drops more rapidly as the incident angle gets larger. It represents that the thermopile with rGO has a smaller view angle. Therefore, the thermopile with rGO can be applied for the application of long-distance temperature measuring.

## 4. Conclusions

In this study, rGO was adopted to improve the sensitivity for the sensing thin film of the IR sensor. A CMOS-MEMS IR sensor with a circular symmetric thermocouple array was designed and fabricated successfully using the TSMC 0.35 μm CMOS process and MEMS post-process. rGO was drop-coated on the floating membrane in the center of the sensor to form a thermal radiation-sensing film or emitter. The thermal radiation measurements including IR absorption and emission, as well as related characteristics of the thermopiles were proceeded with. For the thermopile with rGO, the emissivity of absorption or emission had a significant increase compared with the one without rGO. The emissivity of the thermopile with rGO is higher than 0.95 as expected for the applications of the IR sensor. The sensitivity of the IR sensor without rGO can be improved highly to be 77 %. The frequency response and the thermal time constant of the thermopiles were also analyzed. Further measurements and analyses were arranged, including the noise, S/N ratio and figure of merit, which shows that the proposed CMOS-MEMS IR sensor with rGO has more excellent characteristics than the sensor without rGO. Nevertheless, the process of applying rGO to the sensor by the drop-coating method is difficult to have a high degree of repeatability and consistency. In addition, the measurement results of thermal radiation with different incident angles show that the IR sensor with rGO has a smaller view angle which is suitable for the application of longer-distance measuring.

## Figures and Tables

**Table 1:** Figure of merits of the thermopiles.

Parameter (Unit)	with rGO	without rGO
Responsivity (V/W)	14.522	8.197
Johnson noise (nV/$\sqrt{Hz}$)	13.716	13.716
SNR	364.43	291.54
NEP (W/$\sqrt{Hz}$)	9.45 × 10^−10^	1.67 × 10^−9^
Detectivity (nV/$\sqrt{Hz}$/W)	1.06 × 10^9^	5.99 × 10^8^
Normalized detectivity (cm·$\sqrt{Hz}$/W)	1.59 × 10^7^	9.02 × 10^6^
